# ﻿The molecular phylogenetic position of *Harpagocarpus* (Polygonaceae) sheds new light on the infrageneric classification of *Fagopyrum*

**DOI:** 10.3897/phytokeys.220.97667

**Published:** 2023-02-28

**Authors:** Daozhang Min, Wei Shi, Mohammad Mehdi Dehshiri, Yuting Gou, Wei Li, Kaixuan Zhang, Meiliang Zhou, Bo Li

**Affiliations:** 1 State Key Laboratory of Desert and Oasis Ecology, Xinjiang Institute of Ecology and Geography, Chinese Academy of Sciences, Urumqi 830011, China Jiangxi Agricultural University Nanchang China; 2 Research Centre of Ecological Sciences, College of Agronomy, Jiangxi Agricultural University, Nanchang 330045, China Xinjiang Institute of Ecology and Geography, Chinese Academy of Sciences Urumqi China; 3 The Specimen Museum of Xinjiang Institute of Ecology and Geography, Chinese Academy of Sciences, Urumqi 830011, China The Specimen Museum of Xinjiang Institute of Ecology and Geography, Chinese Academy of Sciences Urumqi China; 4 Department of Biology, Borujerd Branch, Islamic Azad University, Borujerd, Iran Islamic Azad University Borujerd Iran; 5 Institute of Crop Sciences, Chinese Academy of Agricultural Sciences, Beijing 100081, China Institute of Crop Sciences, Chinese Academy of Agricultural Sciences Beijing China

**Keywords:** buckwheat, *cp*DNA, Fagopyreae, morphology, new section

## Abstract

In the context of the molecular phylogeny of Polygonaceae, the phylogenetic positions of most genera and their relationships have been resolved. However, the monotypic genus *Harpagocarpus* has never been included in any published molecular phylogenetic studies. In the present study, we adopt a two-step approach to confirm the phylogenetic placement of *Harpagocarpus* using two datasets: (1) a concatenated dataset of three chloroplast DNA (cpDNA) regions (*matK*, *rbcL* and *trnL-F*) for Polygonaceae and (2) a combined cpDNA dataset of five sequences (*accD*, *matK*, *psbA-trnH*, *rbcL* and *trnL-F*) for *Fagopyrum*. Our analyses confirm the previous hypothesis based on morphological, anatomical and palynological investigations that *Harpagocarpus* is congeneric with *Fagopyrum* and further reveal that *H.snowdenii* (≡ *F.snowdenii*) is sister to the woody buckwheat *F.tibeticum*. Within *Fagopyrum*, three highly supported clades were discovered and the first sectional classification was proposed to accommodate them: sect. Fagopyrum comprises the two domesticated common buckwheat (*F.esculentum* and *F.tataricum*) and their wild relatives (F.esculentumsubsp.ancestrale, *F.homotropicum* and *F.dibotrys*) which are characterised by having large corymbose inflorescences and achenes greatly exceeding the perianth; sect. Tibeticum, including *F.snowdenii* and *F.tibeticum*, is characterised by the achene having appurtenances along the ribs, greatly exceeding the perianth and the perianth accrescent in fruit; sect. Urophyllum contains all other species of which the achenes were completely enclosed in the perianth. This study is very helpful to understand the phylogeny of the *Fagopyrum* and sheds light on the future study of taxonomy, biogeography, diversification and character evolution of the genus.

## ﻿Introduction

Polygonaceae, a family of the flowering plants known as the buckwheat family, can be easily distinguished by its ocrea, orthotropous ovules, trigonal (typically) achenes and quincuncial aestivation (Judd et al. 2007) and is found in almost all ecosystems (Sanchez et al. 2009). Numerous molecular phylogenetic analyses (e.g. Cuénoud et al. (2002); Schäferhoff et al. (2009); Moore et al. (2010); Yang et al. (2015); Walker et al. (2018); Yao et al. (2019); Li et al. (2021)) have provided strong evidence for the monophyly of Polygonaceae and the family’s membership in the FTPP clade of the order Caryophyllales, which also includes the Plumbaginaceae, Polygonaceae, Tamaricaceae and Frankeniaceae, has been securely supported (e.g. Cuénoud et al. (2002); Brockington et al. (2009); Walker et al. (2018)). Since the first large-scale molecular phylogenetic reconstruction of the Polygonaceae in 2003 (Lamb-Frye and Kron 2003), the infrafamilial relationships have gradually been resolved in subsequent studies (e.g. Kim and Donoghue (2008a, b); Kim et al. (2008); Sanchez and Kron (2008, 2009, 2011); Galasso et al. (2009); Sanchez et al. (2009, 2011); Burke et al. (2010); Tavakkoli et al. (2010, 2015); Yurtseva et al. (2010, 2016); Schuster et al. (2011a, b, 2015); Kempton (2012)) and its classification at subfamilial and tribal levels has been significantly improved (Sanchez and Kron 2008; Galasso et al. 2009; Sanchez et al. 2009, 2011; Schuster et al. 2011b, 2015). The majority of genera have been included in previous molecular phylogenetics and their monophyly and circumscription were validated, but a few genera were re-circumscribed, such as *Atraphaxis* L., *Koenigia* L., *Polygonum* L., *Ruprechtia* C.A.Mey. etc. As a result, some new genera were erected, i.e. *Duma* T.M.Schuster (Schuster et al. 2011b), *Salta* Adr.Sanchez and *Magoniella* Adr.Sanchez (Sanchez and Kron 2011), *Bactria* O.V.Yurtseva & E.V.Mavrodiev (Yurtseva et al. 2016), *Persepolium* O.V.Yurtseva & E.V.Mavrodiev (Yurtseva et al. 2017) and several old genera have been reduced, for example, *Aconogonon* (Meisn.) Rchb., *Rubrivena* M.Král and *Emex* Neck. ex Campd. (Schuster et al. 2015), *Parapteropyrum* A.J.Li (Sanchez et al. 2011), *Polygonella* Michx. (Schuster et al. 2011a) etc. However, due to a dearth of materials or insufficient molecular data to date, the systematic positions of two resistant genera, *Harpagocarpus* Hutch. & Dandy and *Eskemukerjea* Malick & Sengupta, have not yet been thoroughly evaluated in molecular analyses (Schuster et al. 2015).

**Figure 1.**
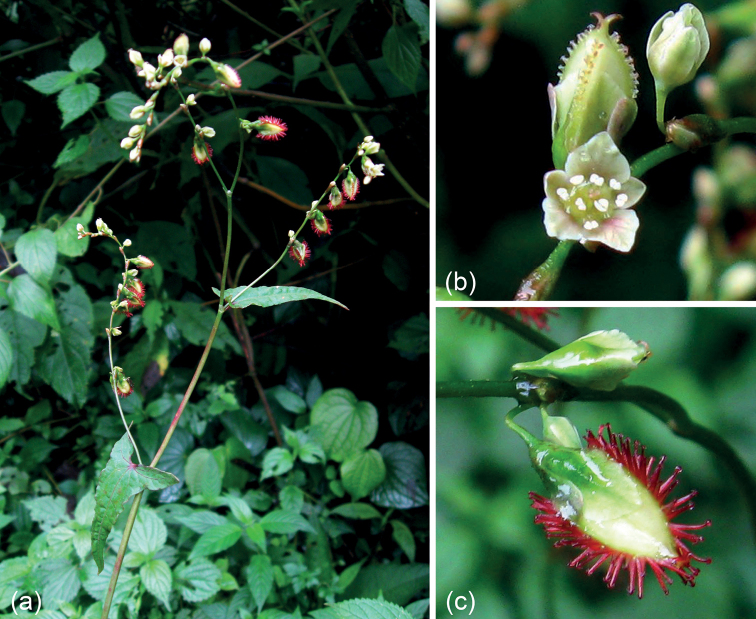
Morphology of *Harpagocarpussnowdenii* Hutch. & Dandy **a** ovate-triangular leaf blades and raceme-like inflorescences **b** an opened flower and a young fruit with minute papillae **c** a mature fruit with long purple setae. (Photographer: Vincent Droissart).

The genus *Harpagocarpus* was established on the basis of its distinct fruit morphology (Hutchinson and Dandy 1926) and contains the sole species, *H.snowdenii* Hutch. & Dandy, which was originally recorded only in Uganda, but now has been reported from Kenya, Tanzania, Rwanda and Cameroon (Ayodele 2003). Jacques-Félix (1946) described *Fagopyrumciliatum* Jacq.-Fél. from Cameroon, but according to Graham (1958), it is merely a synonym of *H.snowdenii*. Due to its unique appurtenances growing along the achene ribs, which are long purple setae with the radially arranged retrorse barbs at the tip of each seta (Fig. 1), *H.snowdenii* is a distinctive species in Polygonaceae (Hutchinson and Dandy 1926).

In the protologue, *Harpagocarpus* was morphologically compared to *Polygonum* L. and *Fagopyrum* Mill., but it was thought to resemble the latter considerably more on its broad cotyledons, large and obviously exerted fruits and the shape and venation of the leaves (Hutchinson and Dandy 1926). On the basis of anatomical similarities, Haraldson (1978) hypothesised that *Harpagocarpus* may be closely related to *Fallopia* Adans. However, Ronse Decraene and Akeroyd (1988) argued against this hypothesis and pointed out that *Harpagocarpus* and *Fagopyrum* share considerable similarity in the morphology of floral characteristics. Hong (1988) further reduced *Harpagocarpus* to a synonym of *Fagopyrum* and proposed the new combination *F.snowdenii* (Hutch. & Dandy) S.P.Hong for *H.snowdenii* after concluding from additional palynological research. Though this treatment has been followed in some literature (e.g. Brandbyge (1993); Friis and Vollesen (1998); Sanchez et al. (2011); de Klerk et al. (2015)), it was, nonetheless, recommended that molecular data be used to confirm the phylogenetic position of *Harpagocarpus* (Schuster et al. 2015).

In the present study, we obtained a few precious pieces of leaf materials of *H.snowdenii* from the specimen *Marshall A.R. WK 374* (detailed information available from: http://legacy.tropicos.org/image/100427626), which provided us an invaluable opportunity to investigate the phylogenetic position of *Harpagocarpus*, based on additional molecular data. We adopted two steps of phylogenetic analyses to infer the generic and specific affinities of *H.snowdenii*. Firstly, we used three chloroplast DNA (cpDNA) markers (*matK*, *rbcL* and *trnL-F*) to present the backbone phylogeny of Polygonaceae and affirmed the position of *Harpagocarpus* in *Fagopyrum*. Subsequently, based on five cpDNA regions (*accD*, *matK*, *psbA-trnH*, *rbcL* and *trnL-F*), we further reconstructed the phylogeny of *Fagopyrum* and clarified the accurate specific relationships of *F.snowdenii* within *Fagopyrum*.

## ﻿Materials and methods

### ﻿Taxon sampling, choice of markers and datasets

We employed *matK*, *rbcL* and *trnL-F* sequences, which have been extensively used in previous studies (e.g. Lamb-Frye and Kron (2003); Sanchez and Kron (2008); Sanchez et al. (2009, 2011); Burke et al. (2010); Schuster et al. (2015)), to generate a concatenated cpDNA dataset (D1) for reconstructing the backbone phylogeny of Polygonaceae. The ingroup taxa were selected from the entire family to cover all recognised tribal clades (Sanchez et al. 2011; Kempton 2012; Schuster et al. 2015) with at least one representative of each genus. A total of 37 genera and 77 species were sampled. *Plumbagoauriculata* Lam. from Plumbaginaceae, which is the sister family of Polygonaceae (Yao et al. 2019; Li et al. 2021), was selected as the outgroup taxon. The source publications or voucher information for all sequences were listed in Suppl. material 1: table S1.

As the analyses of the D1 dataset demonstrated that *Harpagocarpus* is nested within *Fagopyrum*, we designed another dataset (D2) using five cpDNA regions (*accD*, *matK*, *psbA-trnH*, *rbcL* and *trnL-F*), with an expanded sampling of *Fagopyrum* aiming for a more accurate placement of *H.snowdenii* (= *F.snowdenii*). The ingroups of D2 dataset included 33 taxa of *Fagopyrum* covering most of the recognised species in the genus and the outgroup taxon was set as *Pteroxygonumgiraldii* Damm. et Diels according to the results presented in Schuster et al. (2015). Voucher information and GenBank accession numbers for taxa used in the D2 dataset are provided in Suppl. material 1: table S2.

### ﻿DNA extraction, amplification and sequencing

Total genomic DNA was extracted from fresh or silica gel dried leaves following the manufacturer’s specifications of the DNEasy Plant Mini Kit (Qiagen, Valencia, CA, USA). After extraction, the DNA was resuspended in double-distilled water and kept at -40 °C for polymerase chain reaction (PCR). The PCR reactions and amplification protocol followed Schuster et al. (2011a). The amplified products were purified using a PCR Product Purification Kit (Shanghai SBS, Biotech Ltd., China). Sequencing reactions were conducted with the forward and reverse PCR primers using the DYEnamic ET Terminator Cycle Sequencing Kit (Amersham Biosciences, Little Chalfont, Buckinghamshire, U.K.) with an ABI PRISM 3730 automatic DNA sequencer (Shanghai Sangon Biological Engineering Technology & Services Co., Ltd., Shanghai, China). Both strands of the DNA were sequenced with overlapping regions to ensure that each base was unambiguous. Electropherograms were assembled and consensus sequences were generated with Geneious Prime 2022.0.2 platform.

### ﻿Phylogenetic analysis

Sequencher version 5.4.6 (Gene Codes Corporation 2021) was used to evaluate chromatograms for base confirmation and editing contiguous sequences. All DNA sequences were initially aligned using Clustal X version 2.1 (Larkin et al. 2007) and adjusted manually in BioEdit Sequence Alignment Editor version 7.2.1 (Hall 1999).

Phylogenetic analyses were conducted, based on the combined cpDNA dataset D1 and D2. The cpDNA regions were supposedly safe to be combined in phylogenetic analyses (Olmstead and Sweere 1994) because the plastid genome is mostly uniparentally inherited (Soltis and Soltis 1998). The datasets were analysed separately using the methods of Maximum Likelihood (ML) and Bayesian Inference (BI).

ML and BI analyses were carried out using RAxML-HPC2 version 8.2.9 (Stamatakis 2014) and MrBayes version 3.2.2 (Ronquist et al. 2012) as implemented on the CIPRES Science Gateway (Miller et al. 2010), respectively. The ML analysis was performed under the GTRGAMMA model with the bootstrap iterations (-# | -N) set to 1000. The BI analysis was executed with most of the default parameters, but manually setting the following: the best substitution types (Nst) and rate distribution models (rates) that were determined by the jModelTest version 2.1.7 (Darriba et al. 2012), sampling one tree every 3000 generations for 100 million generations, stop early if the convergence diagnostic falls below the stop value 0.001 and show tree probabilities on the 50% majority-rule consensus tree with simple output format.

## ﻿Results

### ﻿Phylogenetic analyses of Polygonaceae

**Figure 2.**
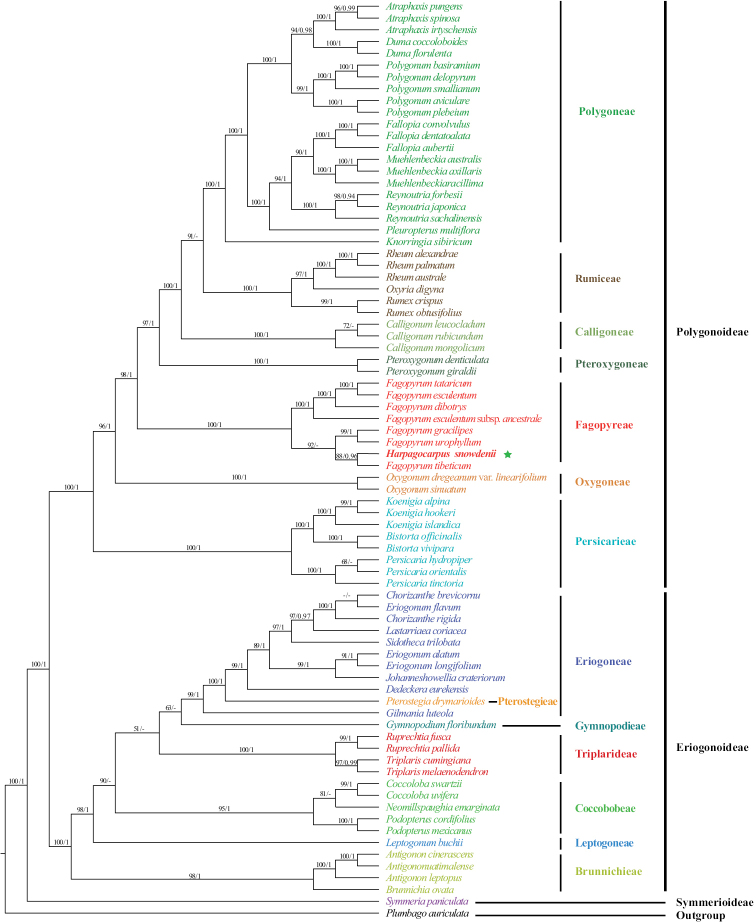
Maximum Likelihood phylogram of Polygonaceae as inferred from analysis of the combined cpDNA dataset of *matK*, *rbcL* and *trnL-F*. Support values ≥ 50% BS or 0.90 PP are displayed above the branches, respectively. The tribal classification of Eriogonoideae followed Sanchez and Kron (2008) and Kempton (2012) and that of Polygoniodeae followed Sanchez et al. (2011) and Schuster et al. (2015). The green star indicates the position of *Harpagocarpussnowdenii*.

The concatenated cpDNA dataset D1 has 78 aligned sequences and comprises 4167 characters (1585 bp for *matK*, 1432 bp for *rbcL* and 1150 bp *trnL-F*, respectively), of which 1756 are variable (42.14%) and 1181 are parsimony-informative (28.34%). The ML and BI analyses, based on dataset D1, generated nearly identical topologies (Suppl. material 1: figs S1, S2); therefore, only the ML tree is presented, with ML bootstrap (BS) and posterior probabilities (PP) values marked on each branch, respectively (Fig. 2). The ingroup (Polygonaceae) is well supported as monophyletic (Fig. 2; BS = 100%, PP = 1.00; all support values follow this order hereafter). Within Polygonaceae, the first branch, represented by *Symmeriapaniculata* Benth., is Symmerioideae which is sister to a large clade comprising Eriogonoideae and Polygonoideae. Within Eriogonoideae, six tribes are recovered with Brunnichieae emerging as the first divergent clade and then subsequently followed by Leptogoneae, Coccobobeae, Triplarideae, Gymnopodieae and Eriogoneae+Pterostegieae. *Pterostegiadrymarioides* Fisch. & C.A.Mey. of Pterostegieae is shown to be nested within Eriogoneae in our analyses. In Polygonoideae, all seven tribes are fully supported as monophyletic (Fig. 2) with Persicarieae, Oxygoneae, Fagopyreae, Pteroxygoneae, Calligoneae and Rumiceae successively sister to the rest. With the inclusion of *Harpagocarpus*, *Fagopyrum* obtained high support values (Fig. 2; 100, 1.00).

### ﻿Phylogenetic analyses of *Fagopyrum*

**Figure 3.**
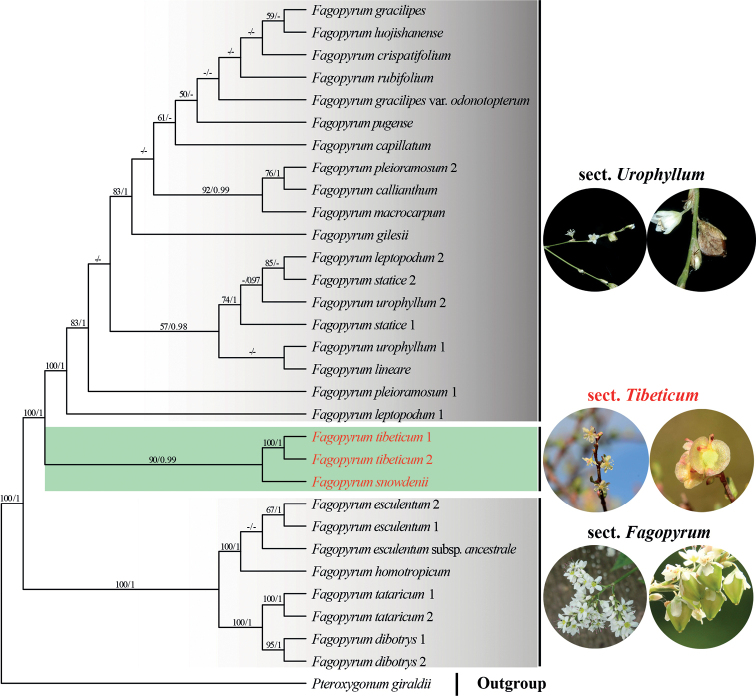
Maximum Likelihood phylogram of *Fagopyrum* as inferred from analysis of the combined cpDNA dataset of *accD*, *matK*, *psbA-trnH*, *rbcL* and *trnL-F*. Support values ≥ 50% BS or 0.90 PP are displayed above the branches, respectively. The two black boxes covered the *cymosum* group and the *urophyllum* group as defined in Yasui and Ohnishi (1998a), respectively. The green box indicates the third group, namely the *tibeticum* group, as revealed in the present study. The representative photos in the circles showing the inflorescences and the fruits of *urophyllum* group, *tibeticum* group and *cymosum* group are *F.urophyllum*, *F.tibeticum* and *F.dibotrys*, respectively. All photos were taken by Bo Li.

The combined dataset D2 has 31 aligned sequences and comprises 6378 characters (1425 bp for *accD*, 2278 bp for *matK*, 513 bp for *psbA-trnH*, 1278 bp for *rbcL* and 883 bp for *trnL-F*), of which 735 are variable (11.52%) and 428 are parsimony-informative (6.71%). ML and BI trees generated from the D2 dataset yielded similar topologies (Suppl. material 1: figs S3, S4); thus, only the ML tree is shown (Fig. 3). In both of the analyses, the monophyly of *Fagopyrum* was strongly supported and three monophyletic subclades were recovered: the first subclade comprises *F.esculentum* Moench, F.esculentumsubsp.ancestrale Ohnishi, *F.homotropicum* Ohnishi, *F.tataricum* (L.) Gaertn. and *F.dibotrys* (D.Don) H.Hara (100, 1.00), the second one is formed by *F.snowdenii* (≡ *Harpagocarpussnowdenii*) and *F.tibeticum* (A.J.Li) Adr.Sanchez & Jan.M.Burke (90, 0.99) and the third includes the remaining taxa of the genus.

## ﻿Discussion

### ﻿Phylogenetic placement of *Harpagocarpus* in Polygonaceae

After 20 years of molecular reconstruction of Polygonaceae (e.g. Kim and Donoghue (2008a); Sanchez and Kron (2008, 2009, 2011); Galasso et al. (2009); Sanchez et al. (2009, 2011); Burke et al. (2010); Tavakkoli et al. (2010, 2015); Yurtseva et al. (2010, 2016, 2017); Schuster et al. (2011a, b, 2015); Tian et al. (2011); Kempton (2012); Fan et al. (2021)), only a few recalcitrant genera, such as *Harpagocarpus* and *Eskemukerjea*, have not been included in molecular analyses and their phylogenetic positions are still unresolved. *Harpagocarpus* is distinct from all other genera of Polygonaceae in having a remarkable fruit that has long setae growing along the ribs with the radially arranged retrorse barbs at the tip of each seta (Hutchinson and Dandy 1926) (Fig. 1). The current study is the first to include the genus *Harpagocarpus* in molecular phylogenetics and it demonstrates in all analyses, based on the cpDNA datasets D1 and D2, that *H.snowdenii*, the sole species of the genus, is deeply nested within *Fagopyrum* (Figs 2, 3). The additional molecular evidence undoubtedly confirms the previous hypothesis that *Harpagocarpus* and *Fagopyrum* are congeneric in respect of morphological, anatomical and palynological investigations (Hong 1988; Ronse Decraene and Akeroyd 1988; Hong et al. 1998).

Morphologically, *H.snowdenii* has sagittate to ovate-triangular leaf blades, seven palmate veins, large and clearly exerted fruits from the persistent tepals and broad cotyledons, which are very similar to those traits presented in *Fagopyrum* species (Hutchinson and Dandy 1926). Anatomical studies showed that *H.snowdenii* and *Fagopyrum* species share a series of similar floral characteristics, such as nectaries which are present as receptacular mamillae behind the stamens, inner stamens which are always linked with two lateral nectaries and cells of the inner tepal epidermis which are rectangular to elongated (Hong 1988; Ronse Decraene and Akeroyd 1988). Palynologically, Ronse Decraene and Akeroyd (1988) emphasised that they observed an identical pollen structure between *Harpagocarpus* and *Fagopyrum* and Hong (1988) further noted that it is hardly possible to find any pollen morphological differences between *Harpagocarpus* and the species of *Fagopyrum*. The pollen of *Fagopyrum* is ovate, tricolpate with narrow furrows and a reticulate surface pattern. The pollen grains of *Harpagocarpus* are slightly smaller than those of *Fagopyrum* species, but they undoubtedly belong to the same pollen type (Hong 1988; Ronse Decraene and Akeroyd 1988). Considering this evidence, Ronse Decraene and Akeroyd (1988) suggested that *H.snowdenii* should probably be included within *Fagopyrum*, perhaps as a separate section and Hong (1988) formally combined *H.snowdenii* as *F.snowdenii*.

It is noteworthy to point out that our molecular analyses not only supported the amalgamation of *Harpagocarpus* with *Fagopyrum*, but also clarified the accurate specific relationships of *F.snowdenii* within *Fagopyrum*, which was stably supported to be a sister of *F.tibeticum* using cpDNA sequences (Figs 2, 3). *F.tibeticum* was originally described in the monotypic genus *Parapteropyrum* A.J.Li as *P.tibeticum* A.J.Li, which is a shrub, endemic to the central Qinghai-Tibetan Plateau of China and is characterised by having fascicled leaves, terminal raceme-like inflorescences, five unequally lobed tepals with the outer two smaller, perianth persistent and accrescent in fruit, three free styles with capitate stigmas and trigonous achenes with broad wings along ribs (Li 1981). *P.tibeticum* was considered to be most similar to *Pteropyrum* Jaub. & Spach in gross morphology (Li 1981), but surprisingly tested to be a member of *Fagopyrum* in molecular phylogenetic studies (Sanchez et al. 2009; Tavakkoli et al. 2010; Tian et al. 2011) and, thus, formally combined in *Fagopyrum* as *F.tibeticum* (Sanchez et al. 2011). The origin of the woody *F.tibeticum* was thought to be a consequence of the large-scale uplift of the Qinghai-Tibetan Plateau which not only promoted continental species radiation, but also the secondary feature of woodiness in a few herbaceous lineages in response to strong selection pressures (Tian et al. 2011).

The inclusion of *F.tibeticum* in *Fagopyrum* has updated our knowledge of morphology in the genus, but now, the sister relationships between *F.snowdenii* and *F.tibeticum*, revealed in our molecular analyses, would not only further expand the morphological variation of *Fagopyrum*, but also shed light on the thinking of the biogeographical origin of the genus, because *F.snowdenii* is the only species of *Fagopyrum* distributed in Africa, while all other congeneric taxa occur mainly in East Asia. Jacques-Félix (1946) suggested that *Fagopyrum* perhaps entered Africa via a Middle Asian pathway during the Quaternary-periglacial period, just like other genera with both Afromontane and Central Asian representatives, such as *Cicer* L. and *Colutea* L. (Chapman and White 1970). However, de Klerk et al. (2015) stated that long-distance transport of pollen grains of *F.snowdenii* from Asia to Africa seems unlikely, but alternatively, they found out there are indications from pollen and macrofossils that a wild *Fagopyrum* ancestor may have been widespread in western Eurasia during the Late Tertiary and the Pleistocene Ice-Ages and became extinct afterwards. *F.snowdenii* may represent the only surviving African lineage that split from the wild widespread *Fagopyrum* ancestor.

### ﻿Infrageneric relationships within *Fagopyrum*

*Fagopyrum* is a small genus comprised of ca. 25 species according to the most updated classification (Ohsako and Li 2020). The genus is economically important and well known for containing two domesticated common buckwheat, i.e. *F.esculentum* and *F.tataricum* which have been widely cultivated in Australia, Asia, Europe and North America for producing gluten-free grains (Li and Hong 2003). Geographically, most of the wild species of *Fagopyrum* are mainly distributed in mountainous regions of southwest China, a few are endemic to the south-eastern edge of the Qinghai-Tibetan Plateau (Ohnishi and Matsuoka 1996; Ohnishi 1998; Li and Hong 2003) and only the *F.snowdenii* confirmed in the present study is occurring in Africa (Hutchinson and Dandy 1926; Ayodele 2003). Eastern Tibet to western Sichuan of China was indicated to be the birthplace of the two cultivated common buckwheat in the AFLP (amplified fragment length polymorphism) analysis (Konishi et al. 2005). Taxonomically, *Fagopyrum* was separated from the large and heterogenous Linnaeus’s genus *Polygonum* L. (Miller 1754) and has long been treated as a section of *Polygonum* (e.g. Meisner (1856); Samuelsson (1929); Steward (1930)) or considered to be an independent genus, but closely related to *Polygonum* (e.g. Dammer (1894); Gross (1913); Hedberg (1946); Haraldson (1978); Ronse Decraene and Akeroyd (1988)). In the context of the molecular phylogeny of Polygonaceae, *Fagopyrum* was not only supported as a monophyletic genus, but also indicated to represent an isolated tribal clade in the subfamily Polygonoideae (Sanchez et al. 2011; Schuster et al. 2015). Morphologically and anatomically, *Fagopyrum* could be distinguished from other genera of Polygonoideae by having large conduplicate cotyledons and/or embryos in the central region in achene (Dammer 1894; Gross 1913; Nakai 1926; Chapman and White 1970; Sanchez et al. 2011).

Within *Fagopyrum*, two groups have been recognised in classical taxonomy, based on the morphology of inflorescence and the achene size: one group was mainly represented by *F.cymosum* (Trevir.) Meisn. (= *F.dibotrys*), *F.esculentum* and *F.tataricum* and characterised by having corymbose inflorescences with many branching and dense flowers and the achene greatly exceeding the perianth, while the other group is composed of other species (including *F.urophyllum* (Bureau & Franch.) H.Gross) having raceme-like inflorescences with sparse flowers and the achene completely enclosed in perianth (Gross 1913; Roberty and Vautier 1964; Ohnishi and Matsuoka 1996) (Fig. 3). These two groups are mostly concordant with the *cymosum* group and the *urophyllum* group defined by Yasui and Ohnishi (1998a) in molecular phylogenetic analyses using DNA sequences of the nuclear internal transcribed spacer (nrITS) and cpDNA region *rbcL*-*accD*. Other molecular studies, no matter using isozyme variability and RFLP (Ohnishi and Matsuoka 1996), cpDNA sequences (Yasui et al. 1998; Ohsako et al. 2001; Jin et al. 2018), nuclear genes or regions (Yasui and Ohnishi 1998b; Nishimoto et al. 2003) and complete plastomes (Fan et al. 2021; Li et al. 2022), all clearly indicated that the *cymosum* group and the *urophyllum* group are both monophyletic clades.

In our present analyses, the above-mentioned two clades were recovered too, but the third clade, formed by *F.snowdenii* and *F.tibeticum*, was discovered, which is sister to the ‘Urophyllum’ clade (Fig. 3). We failed to generate any nuclear sequences from the specimen sample of *F.snowdenii*; thus, we could not test the sister relationships between *F.snowdenii* and *F.tibeticum*, as well as the sister relationships between *F.snowdenii* + *F.tibeticum* clade and the ‘Urophyllum’ clade in nuclear analysis. However, when only *F.tibeticum* was included in the ITS analysis, the topology of the phylogenetic tree is similar to that yielded from the combined cpDNA dataset, in which *F.tibeticum* is sister to the ‘Urophyllum’ clade clade (Tian et al. 2011). Considering the sister relationships between *F.snowdenii* and *F.tibeticum* could be additionally supported by morphological and palynological evidence, such as raceme-like inflorescences, unequal tepals with the outer two smaller, perianth accrescent in fruit, large achenes greatly exceeding the perianth, special appurtenances (either wings or setae) growing along the fruit ribs and smaller pollen grains than the other *Fagopyrum* species (Hutchinson and Dandy 1929; Ronse Decraene and Akeroyd 1988; Hong 1995), we believe that *F.snowdenii* and *F.tibeticum* represent a separate clade in *Fagopyrum*. Future analyses, based on more comprehensive sampling and using nuclear sequences data, may further confirm or update the infrageneric relationships of *Fagopyrum* as inferred in this study. As far as the current results are concerned, a sectional classification for *Fagopyrum* is here proposed, based on the differentiation of gross morphology in the three clades, which is the first infrageneric classification of the genus.

### ﻿Taxonomic treatment

#### ﻿ Fagopyrum

Taxon classificationPlantaeCaryophyllalesPolygonaceae

Mill., Gard. Dict. Abr.

FF88D72C-2405-5FE1-8728-52CC750C8CBC

Fagopyrum Mill., Gard. Dict. Abr., ed. 4, 495. 1754 [≡ Polygonumsect.Fagopyrum (Mill.) Meisn., Monogr. Polyg. 43, 61. 1826.] – Type: Fagopyrumesculentum Moench (≡ Polygonumfagopyrum L.).
= Harpagocarpus Hutch. & Dandy, Bull. Misc. Inform. Kew. 364. 1926 – Type: Harpagocarpussnowdenii Hutch. & Dandy [≡ Fagopyrumsnowdenii (Hutch. & Dandy) S.P.Hong].
= Parapteropyrum A.J.Li, Acta Phytotax. Sin. 19: 330. 1981 – Type: Parapteropyrumtibeticum A.J.Li [≡ Fagopyrumtibeticum (A.J.Li) Adr.Sanchez & Jan.Burke].

#### ﻿ Fagopyrum sect. Fagopyrum

Taxon classificationPlantaeCaryophyllalesPolygonaceae

850B40D3-92E3-59CF-91DB-28396A7F7AA5

##### Type.

*Fagopyrumesculentum* Moench. (≡ *Polygonumfagopyrum* L.).

##### Diagnosis.

This section is characterised by having large corymbose inflorescences with many branches and dense flowers and large achenes greatly exceeding the persistent perianth.

##### Species.

*F.dibotrys*, F.esculentumsubsp.esculentum, F.esculentumsubsp.ancestrale, *F.homotropicum* and *F.tataricum*.

##### Distribution.

Bhutan, India, Myanmar, Nepal, Pakistan, Thailand and Vietnam of southern and south-eastern Asia and southern and south-western China.

#### ﻿ Fagopyrum sect. Tibeticum

Taxon classificationPlantaeCaryophyllalesPolygonaceae

Bo Li & M.L.Zhou sect. nov.

F19BE93D-AD33-51E9-8E0E-E79D27778999

urn:lsid:ipni.org:names:77315008-1

##### Type.

*Fagopyrumtibeticum* (A.J.Li) Adr. Sanchez & Jan. Burke (≡ *Parapteropyrumtibeticum* A.J.Li).

##### Diagnosis.

The new section is characterised by having raceme-like inflorescences with sparse flowers, large achenes with appurtenances (wings or setae) along the ribs and greatly exceeding the perianth and persistent perianth accrescent in fruit.

##### Species.

*F.snowdenii* and *F.tibeticum*.

##### Distribution.

Cameroon, Kenya, Rwanda, Tanzania and Uganda of Africa (*F.snowdenii*) and Tibet of south-western China (*F.tibeticum*).

#### ﻿ Fagopyrum sect. Urophyllum

Taxon classificationPlantaeCaryophyllalesPolygonaceae

Bo Li & M.L.Zhou sect. nov.

17A4EA9A-7716-56A5-86E2-3A8FEE18EF91

urn:lsid:ipni.org:names:77315009-1

##### Type.

*Fagopyrumurophyllum* (Bureau & Franch.) H.Gross (≡ *Polygonumurophyllum* Bureau & Franch.).

##### Diagnosis.

This new section is characterised by having raceme-like, spicate, capitate or paniculate inflorescences with mostly sparse or rarely dense flowers and achenes completely enclosed in the persistent perianth.

##### Species.

*F.callianthum* Ohnishi, *F.capillatum* Ohnishi, *F.caudatum* (Sam.) A.J.Li, *F.crispatifolium* J.L.Liu, *F.densovillosum* J.L.Liu, *F.gilesii* (Hemsl.) Hedberg, *F.gracilipedoides* Ohsako & Ohnishi, *F.gracilipes* (Hemsl.) Dammer, *F.jinshaense* Ohsako & Ohnishi, F.leptopodum(Diels)Hedbergvar.leptopodum, F.leptopodumvar.grossii (Lévl.) Lauener & D.K.Ferguson, *F.lineare* (Sam.) Haraldson, *F.longistylum* M.L.Zhou & Y.Tang, *F.longzhoushanense* J.R.Shao, *F.luojishanense* J.R.Shao, *F.macrocarpum* Ohsako & Ohnishi, *F.pleioramosum* Ohnishi, *F.pugense* Y.Tang, *F.qiangcai* D.Q.Bai, *F.rubifolium* Ohsako & Ohnishi, *F.statice* H.Gross, *F.urophyllum* (Bureau & Franch.) H.Gross, *F.wenchuanense* J.R.Shao.

##### Distribution.

Guizhou, Sichuan and Yunnan Provinces of southwest China.

### ﻿Identification keys to three sections of *Fagopyrum*

**Table:** 

1	Achenes completely enclosed in the perianth	**sect. Urophyllum**
–	Achenes greatly exceeding the perianth	**2**
2	Raceme-like inflorescences with sparse flowers and achenes having appurtenances (wings or setae) along the ribs	**sect. Tibeticum**
–	Corymbose inflorescences with dense flowers and achenes without appurtenances	**sect. Fagopyrum**

## Supplementary Material

XML Treatment for Fagopyrum

XML Treatment for Fagopyrum sect. Fagopyrum

XML Treatment for Fagopyrum sect. Tibeticum

XML Treatment for Fagopyrum sect. Urophyllum

## Supplementary materials

Supplementary material 1 Supplementary information Data type tables, figures (Pdf file) Explanation note Taxa, GenBank accession numbers of DNA sequences with their vouchers or source of publication used in the molecualr dataset and phtlogenetic trees generated from BI and ML analyses. https://binary.pensoft.net/file/816723This dataset is made available under the Open Database License (http://opendatacommons.org/licenses/odbl/1.0/). The Open Database License (ODbL) is a license agreement intended to allow users to freely share, modify, and use this Dataset while maintaining this same freedom for others, provided that the original source and author(s) are credited.Daozhang Min, Wei Shi, Mohammad Mehdi Dehshiri, Yuting Gou, Wei Li, Kaixuan Zhang, Meiliang Zhou, Bo Li
