# Supplementary material for: ﻿The molecular phylogenetic position of Harpagocarpus (Polygonaceae) sheds new light on the infrageneric classification of Fagopyrum
Source: PhytoKeys. 2023 Feb 28;220:109–26. doi: 10.3897/phytokeys.220.97667 (PMC10209611; doi:10.3897/phytokeys.220.97667)

**Table S1.** Taxa, GenBank accession numbers of DNA sequences with their vouchers or source of publication used in the combined cpDNA dataset D1 of Polygonaceae. Newly sequenced taxa are shown in bold, and missing data are indicated by a dash (-).

| **Taxa** | ***matK*** | **Voucher/Reference** | ***rbcL*** | **Voucher/Reference** | ***trnL-F*** | **Voucher/Reference** |
|---|---|---|---|---|---|---|
| *Antigonon cinerascens* | HM137385 | Burke et al. (2010) | HM137363 | Burke et al. (2010) | - | - |
| *Antigonon guatimalense* | FJ154491 | Luckow 4034 (BH) | FJ154449 | Luckow 4034 (BH) | - | - |
| *Antigonon leptopus* | MH286313 | M. J. Moore 1811 (OC) | MH286313 | M. J. Moore 1811 (OC) | MH286313 | M. J. Moore 1811 (OC) |
| *Atraphaxis irtyschensis* | MG878984 | Duan. (2018) | MG878984 | Duan. (2018) | MG878984 | Duan. (2018) |
| *Atraphaxis pungens* | EU840454 | J.Q.Liu rzml | EU840286 | J.Q.Liu rzml | EU840538 | J.Q.Liu rzml |
| *Atraphaxis spinosa* | EU840453 | J.Q.Liu cml | EU840285 | J.Q.Liu cml | EU840537 | J.Q.Liu cml |
| *Bistorta officinalis* | NC_065784 | Guo et al. (2021) | NC_065784 | Guo et al. (2021) | NC_065784 | Guo et al. (2021) |
| *Bistorta vivipara* | EU840456 | J.Q.Liu zyl2 | EU840288 | J.Q.Liu zyl2 | EU840540 | J.Q.Liu zyl2 |
| *Brunnichia ovata* | EF437990 | Sanchez and Kron.2007 | FJ154451 | Meloche s.n. (ARS-USDA) | JQ352627 | Kempton (2012) |
| *Calligonum leucocladum* | NC_053260 | IBSC:TLF-109 | NC_053260 | IBSC:TLF-109 | NC_053260 | IBSC:TLF-109 |
| *Calligonum mongolicum* | NC_053261 | IBSC:Q-202 | NC_053261 | IBSC:Q-202 | NC_053261 | IBSC:Q-202 |
| *Calligonum rubicundum* | EU840493 | J.Q.Liu hpsgz | EU840325 | J.Q.Liu hpsgz | EU840551 | J.Q.Liu hpsgz |
| *Chorizanthe brevicornu* | EF437991 | Sanchez & Kron (2007) | EF437974 | Sanchez & Kron (2007) | JQ352630 | Kempton (2012) |
| *Chorizanthe rigida* | EF437993 | Sanchez & Kron (2007) | EF437975 | Sanchez & Kron (2007) | JQ352647 | Kempton (2012) |
| *Coccoloba swartzii* | KJ012532 | 0132456805 | KJ082221 | 0132456805 | MT327014 | Koenemann & Burke 2020 |
| *Coccoloba uvifera* | KJ012536 | 0118737148 | KJ082225 | 0118737148 | JQ352754 | Kempton (2012) |
| *Dedeckera eurekensis* | EF437997 | Sanchez & Kron (2007) | EF437976 | Sanchez & Kron (2007) | JQ352654 | Kempton (2012) |
| *Duma florulenta* | JF831265 | Schuster. (2011) | - | - | JF831298 | Schuster. (2011) |
| *Duma coccoloboides* | JF831264 | Schuster. (2011) | - | - | JF831297 | Schuster. (2011) |
| *Eriogonum alatum* | EF437998 | Sanchez & Kron (2007) | EF437977 | Sanchez & Kron (2007) | JQ352614 | Kempton (2012) |
| *Eriogonum flavum* | AY042584 | Cuenoud et al. (2001) | MG248611 | CCDB-21230-B05 | JQ352670 | Kempton (2012) |
| *Eriogonum longifolium* | KJ772763 | Abbott 22683 (FLAS) | KJ773487 | Abbott 22683 (FLAS) | JQ352696 | Kempton (2012) |

| | | | | | | |
|---|---|---|---|---|---|---|
| *Fagopyrum dibotrys* | KP404630 | Yang et al. (2015) | KP404630 | Yang et al. (2015) | KP404630 | Yang et al. (2015) |
| *Fagopyrum esculentum* | EU840460 | J.Q.Liu rh6270 | EU840292 | J.Q.Liu rh6270 | EU840543 | J.Q.Liu rh6270 |
| *Fagopyrum esculentum* subsp. *ancestrale* | EU254477 | Logacheva et al. (2008) | EU254477 | Logacheva et al. (2008) | EU254477 | Logacheva et al. (2008) |
| *Fagopyrum gracilipes* | MK435798 | TianXJ0160 | MN204820 | TianXJ0160 | EU024787 | Y.T.Hou080(SDNU) |
| *Fagopyrum tataricum* | MN273657 | LJQ-2008-GN-205 | MN204821 | LJQ-2008-GN-205 | KP966729 | BT13 |
| *Fagopyrum tibeticum* | - | - | JQ009278 | Sun & zhang. (2011) | JQ009296 | Sun & zhang. (2011) |
| *Fagopyrum urophyllum* | OK054490 | Zhou (2021) | OK054490 | Zhou (2021) | OK054490 | Zhou (2021) |
| *Fallopia aubertii* | EU840492 | J.Q.Liu mtl4 | EU840324 | J.Q.Liu mtl4 | EU840550 | J.Q.Liu mtl4 |
| *Fallopia convolvulus* | EU024770 | Y.T.Hou036(SDNU) | HM357893 | CPU:L.Q. Zhao 0893 | EU024782 | Y.T.Hou036(SDNU) |
| *Fallopia dentatoalata* | EU024769 | F.Z.Li4069(SDNU) | HM357888 | CPU:L.Q. Zhao 0891 | EU024775 | F.Z.Li4069(SDNU) |
| *Gilmania luteola* | EF438010 | Sanchez & Kron (2007) | HM137378 | Burke et al. (2010) | JQ352672 | Kempton (2012) |
| *Gymnopodium floribundum* | GQ206197 | Sanchez et al. (2009) | GQ206220 | Sanchez et al. (2009) | JQ352688 | Kempton (2012) |
| ***Harpagocarpus snowdenii*** | ● | Marshall WK374 (MO) | ● | Marshall WK374 (MO) | ● | Marshall WK374 (MO) |
| *Johanneshowellia crateriorum* | EF438011 | Sanchez & Kron (2007) | EF437986 | Sanchez & Kron (2007) | JQ352689 | Kempton (2012) |
| *Koenigia alpina* | HM357917 | CPU:L.Q. Zhao 0892 | HM357891 | CPU:L.Q. Zhao 0892 | KR537738 | TMS14-07 (MEL) |
| *Koenigia hookeri* | EU840457 | J.Q.Liu 1733 | EU840289 | J.Q.Liu 1733 | EU840541 | J.Q.Liu 1733 |
| *Koenigia islandica* | EU840455 | J.Q.Liu bdl3 | EU840287 | J.Q.Liu bdl3 | EF653789 | Lutz s.n (YU) |
| *Knorringia sibiricum* | EU024771 | F.Z.Li0182(SDNU)/D1340 | JN234982 | FanDM-020 | JN235064 | FanDM-020 |
| *Lastarriaea coriacea* | KC977545 | Kostikova et al. (2013) | - | - | JQ352693 | Kempton (2012) |
| *Leptogonum buchii* | GQ206199 | Sanchez et al. (2009) | GQ206223 | Sanchez et al.2009 | JQ352611 | Kempton (2012) |
| *Muehlenbeckia australis* | NC_059029 | CHR655506 | NC_059029 | CHR655506 | NC_059029 | CHR655506 |
| *Muehlenbeckia axillaris* | NC_059030 | CANB873389 | NC_059030 | CANB873389 | NC_059030 | CANB873389 |
| *Muehlenbeckia gracillima* | NC_059031 | CANB615519 | NC_059031 | CANB615519 | NC_059031 | CANB615519 |
| *Neomillspaughia emarginata* | GQ206201 | Sanchez et al. (2009) | GQ206225 | Sanchez et al.2009 | - | - |
| *Oxygonum dregeanum* var. | JN161150 | PRE:P. Winter 6662 | - | - | JN161140 | PRE:P. Winter 6662 |

| | | | | | | |
|---|---|---|---|---|---|---|
| *linearifolium* | | | | | | |
| *Oxygonum sinuatum* | KR734580 | C6_K1215_Oxygonium_sp | KR736460 | A6_K1245_Oxygonum | KR737905 | C6_K1215_Oxygonium_sp |
| *Oxyria digyna* | EU840459 | J.Q.Liu 2087 | EU840291 | J.Q.Liu 2087 | JN235068 | FanDM-058 |
| *Persicaria hydropiper* | EF653728 | Kim & Ma Ch-Ko-37 (YU) | EF653780 | Kim & Ma Ch-Ko-37 (YU) | EF653806 | Kim & Ma Ch-Ko-37 (YU) |
| *Persicaria orientalis* | NC_065785 | Guo et al. (2021) | NC_065785 | Guo et al. (2021) | NC_065785 | Guo et al. (2021) |
| *Persicaria tinctoria* | KJ939218 | YC0390MT02 | KJ939248 | YC0390MT02 | EU197048 | Kim Ch-Ko-88 (YU) |
| *Pleuropterus multiflora* | MK330002 | XPALSD-368(KUN) | MK330002 | XPALSD-368(KUN) | MK330002 | XPALSD-368(KUN) |
| *Plumbago auriculata* | MK397887 | 14CS9234 (KUN) | MK397887 | 14CS9234 (KUN) | MK397887 | 14CS9234 (KUN) |
| *Podopterus cordifolius* | FJ154494 | Burke 30 (BH) | FJ154455 | Burke 30 (BH) | - | - |
| *Podopterus mexicanus* | KM219808 | UNAM:PB-CIECO 1674 | KU176139 | UNAM:PB-CIECO 1674 | - | - |
| *Polygonum aviculare* | EF653710 | Sultan & Heschel s.n (YU) | EF653761 | Sultan & Heschel s.n (YU) | EF653787 | Sultan & Heschel s.n (YU) |
| *Polygonella basiramium* | KY607313 | FLAS:Cox LWR74 | KX397903 | FLAS:Cox LWR74 | JN161141 | FLAS:A. Cox LWR74, H. Loring |
| *Polygonum delopyrum* | KJ773033 | Abbott 25190 (FLAS) | KJ773777 | Abbott 25190 (FLAS) | JN161142 | FLAS:A. Miller 122 |
| *Polygonum plebeium* | MH768005 | TuTY1484 | MH767706 | TuTY1484 | EU109598 | Wei_and_Ze.2007 |
| *Polygonum smallianum* | KY607314 | FLAS:Orzell 21017 | KX397904 | FLAS:Orzell 21017 | JN161143 | FLAS:J. Larsen s.n. |
| *Pterostegia drymarioides* | GQ206206 | Sanchez et al. (2009) | GQ206229 | Sanchez et al. (2009) | JQ352725 | Kempton (2012) |
| *Pteroxygonum denticulata* | HM357915 | CPU:X. H. Meng 0942 | HM357889 | CPU:X. H. Meng 0942 | EU586187 | CY |
| *Pteroxygonum giraldii* | GQ206207 | Sanchez et al. (2009) | GQ206230 | Sanchez et al. (2009) | EU402464 | Y1 |
| *Reynoutria forbesii* | KJ862994 | Park & Bhandari. (2014) | KJ863208 | Park & Bhandari. (2014) | KJ887291-KJ886970 | Park & Bhandari. (2014) |
| *Reynoutria japonica* | EU024772 | F.Z.Li021028(SDNU) | JF950004 | P6894 | EU024786 | F.Z.Li021028(SDNU) |
| *Reynoutria sachalinensis* | NC_047446 | Raman (2019) | NC_047446 | Raman (2019) | NC_047446 | Raman (2019) |
| *Rheum palmatum* | EU840482 | J.Q.Liu 1075 | EU840314 | J.Q.Liu 1075 | AY566453 | 2082 |
| *Rheum australe* | EU840477 | J.Q.Liu 1101 | EU840309 | J.Q.Liu 1101 | AY566459 | J.Q.Liu 1101 |
| *Rheum alexandrae* | EU840485 | J.Q.Liu 2051 | EU840317 | J.Q.Liu 2051 | KF586493 | 0950 |

| | | | | | | |
|---|---|---|---|---|---|---|
| *Rumex crispus* | EU840458 | J.Q.Liu zysm1 | EU840290 | J.Q.Liu zysm1 | EU840542 | J.Q.Liu zysm1 |
| *Rumex obtusifolius* | HM851086 | BM 2008/374 | HM850322 | BM 2008/374 | KM385510 | Meyer et al. (2014) |
| *Ruprechtia fusca* | FJ154496 | Pendry 868 (E) | FJ154457 | Pendry 868 (E) | KU172549 | MEXU-1388068 |
| *Ruprechtia pallida* | HM137403 | Burke et al. (2010) | HM137383 | Burke et al. (2010) | JQ352730 | Kempton (2012) |
| *Sidotheca trilobata* | MF963694 | RSA773164 | MF963350 | RSA773164 | JQ352746 | Kempton (2012) |
| *Symmeria paniculata* | MH286353 | L. Catarino & Q. Bancessi 529 (WAG) | MH286353 | L. Catarino & Q. Bancessi 529 (WAG) | MH286353 | L. Catarino & Q. Bancessi 529 (WAG) |
| *Triplaris cumingiana* | GQ206210 | Sanchez et al. (2009) | GQ206236 | Sanchez et al. (2009) | MT327021 | Koenemann & Burke 2020 |
| *Triplaris melaenodendron* | JQ588856 | BioBot00791 | JQ593540 | BioBot00791 | JQ352747 | Kempton (2012) |

**Table S2.** Taxa, GenBank accession numbers of DNA sequences with their vouchers or source of publication used in the combined cpDNA dataset D2 of *Fagopyrum*. Newly sequenced taxa are shown in bold, and missing data are indicated by a dash (-).

| Taxa | *accD* | Voucher/Reference | *matK* | Voucher/Reference | *psbA-trnH* | Voucher/Reference | *rbcL* | Voucher/Reference | *trnL-F* | Voucher/Reference |
|---|---|---|---|---|---|---|---|---|---|---|
| *F. callianthum* | AB000302 | Yasui and Ohnishi (1998) | AB026329 | Ohsako and Ohnishi (2000) | - | - | AB000302 | Yasui and Ohnishi (1998) | - | - |
| *F. capillatum* | AB000303 | Yasui and Ohnishi (1998) | AB026321 | Ohsako and Ohnishi (2000) | MT668930 | Cheng (2020) | AB000303 | Yasui and Ohnishi (1998) | - | - |
| *F. crispatifolium* | - | - | JF829975 | Wang (2011) | JQ807567 | P.T. Zheng 02 | - | - | - | - |
| *F. dibotrys* 1 | KY275181 | Shao et al. (2016) | KY275181 | Shao et al. (2016) | EU554044 | Chen et_al. (2008) | KY275181 | Shao et al. (2016) | KY275181 | Shao et al. (2016) |
| *F. dibotrys* 2 | KP404630 | Yang et al. (2015) | KP404630 | Yang et al. (2015) | KP404630 | Yang et al. (2015) | KP404630 | Yang et al. (2015) | KP404630 | Yang et al. (2015) |
| *F. esculentum* subsp. *ancestrale* | EU254477 | Logacheva (2008) | EU254477 | Logacheva (2008) | EU254477 | Logacheva (2008) | EU254477 | Logacheva (2008) | EU254477 | Logacheva (2008) |
| *F. esculentum* 1 | EU840418 | J.Q.Liu rh6270 | EU840460 | J.Q.Liu rh6270 | JQ807581 | P.T. Zheng 16 | EU840460 | J.Q.Liu rh6270 | EU840460 | J.Q.Liu rh6270 |
| *F. esculentum* 2 | AB000309 | Yasui and Ohnishi (1998) | EF653711 | Kim & Kim Ch-Ko-96 (YU) | EF653736 | Kim & Kim Ch-Ko-96 (YU) | AB000309 | Yasui and Ohnishi (1998) | EF653788 | Kim & Kim Ch-Ko-96 (YU) |
| *F. gilesii* | AB056689 | Ohsako et_al. (2001) | AB086257 | Ohsako,T.2002 | KY206923 | Wu,L.-Y.2016 | AB056689 | Ohsako et_al. (2001) | - | - |
| *F. gracilipes* | AB000311 | Yasui and Ohnishi (1998) | MK435798 | TianXJ0160 | JQ807568 | P.T. Zheng 03 | MK435798 | TianXJ0160 | EU024787 | Y.T.Hou080(SDNU) |
| *F. gracilipes* var. *odontopterum* | - | - | JF829974 | Wang (2011) | JQ807566 | P.T. Zheng 01 | - | - | - | - |
| *F. homotropicum* | AB000312 | Yasui and Ohnishi (1998) | - | - | - | - | AB000312 | Yasui and Ohnishi (1998) | - | - |
| *F. leptopodum* 1 | OK054491 | Zhou (2021) | OK054491 | Zhou (2021) | OK054491 | Zhou (2021) | OK054491 | Zhou (2021) | OK054491 | Zhou (2021) |
| *F. leptopodum* 2 | AB000313 | Yasui and Ohnishi (1998) | ● | LB0198 | ● | LB0198 | ● | LB0198 | ● | LB0198 |
| *F. lineare* | AB000314 | Yasui and Ohnishi (1998) | MT668922 | Cheng (2020) | JQ807573 | P.T. Zheng 08 | AB000314 | Yasui and Ohnishi (1998) | - | - |
| *F. luojishanense* | KY275182 | Wang et al. (2016) | KY275182 | Wang et al. (2016) | KY275182 | Wang et al. (2016) | KY275182 | Wang et al. (2016) | KY275182 | Wang et al. (2016) |
| *F. macrocarpum* | AB056687 | Ohsako et_al. (2001) | MT668921 | Cheng (2020) | MT668931 | Cheng (2020) | AB056687 | Ohsako et_al. (2001) | - | - |

| | | | | | | | | | | |
|---|---|---|---|---|---|---|---|---|---|---|
| *F. pleioramosum* 1 | AB000315 | Yasui and Ohnishi (1998) | AB026324 | Ohsako and Ohnishi (2000) | - | - | AB000315 | Yasui and Ohnishi (1998) | - | - |
| *F. pleioramosum* 2 | AB056688 | Ohsako et_al. (2001) | AB026323 | Ohsako and Ohnishi (2000) | - | - | AB056688 | Ohsako et_al. (2001) | - | - |
| *F. pugense* | - | - | JF829976 | Wang (2011) | JQ807569 | P.T. Zheng 04 | - | - | - | - |
| *F. rubifolium* | AB056686 | Ohsako et_al. (2001) | MT668924 | Cheng (2020) | MT668929 | Cheng (2020) | AB056686 | Ohsako et_al. (2001) | - | - |
| *F. statice* 1 | AB000316 | Yasui and Ohnishi (1998) | AB026313 | Ohsako and Ohnishi (2000) | KY206927 | Wu,L.-Y.2016 | AB000316 | Yasui and Ohnishi (1998) | - | - |
| *F. statice* 2 | AB000317 | Yasui and Ohnishi (1998) | AB026309 | Ohsako and Ohnishi (2000) | - | - | AB000317 | Yasui and Ohnishi (1998) | - | - |
| *F. tataricum* 1 | - | - | - | - | ● | LB0362 | ● | LB0362 | ● | LB0362 |
| *F. tataricum* 2 | AB000319 | Yasui.1997 | MN273657 | LJQ-2008-GN-205 | JQ807577 | P.T. Zheng 12 | MN204821 | LJQ-2008-GN-205 | KP966729 | BT13 |
| *Fagopyrum urophyllum* 1 | - | - | ● | LB0809 | ○ | ○ | ● | LB0809 | ● | LB0809 |
| *F. urophyllum* 2 | OK054490 | Zhou (2021) | OK054490 | Zhou (2021) | OK054490 | Zhou (2021) | OK054490 | Zhou (2021) | OK054490 | Zhou (2021) |
| *F. snowdenii* | - | - | ● | Marshall WK374 (MO) | ● | Marshall WK374 (MO) | ● | Marshall WK374 (MO) | ● | |
| *F. tibeticum* 1 | JN187097 | Ljq07232 | - | - | JQ009240 | Sun and zhang (2011) | JQ009278 | Sun and zhang (2011) | JQ009296 | Sun and zhang (2011) |
| *F. tibeticum* 2 | - | - | ● | LB0787 | ● | LB0787 | ● | LB0787 | ● | LB0787 |
| *Pteroxygonum giraldii* | - | - | GQ206207 | Sanchez et al. (2009) | - | - | GQ206230 | Sanchez et al. (2009) | EU402464 | Y1 |

**Figure S1.** BI tree of Polygonaceae based on the combined cpDNA dataset of matK, rbcL, and trnL-F.

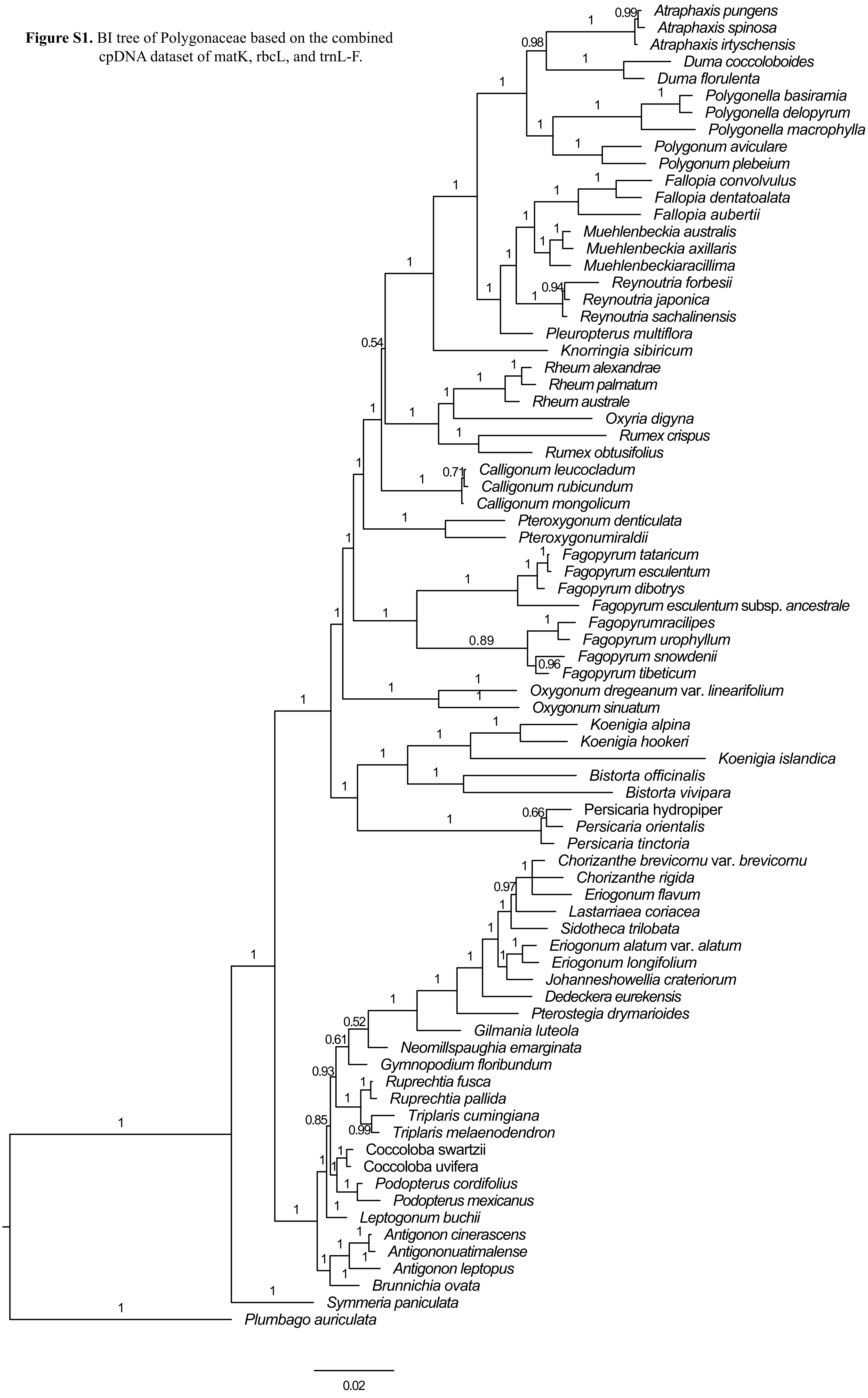

**Figure S2.** ML tree of Polygonaceae based on the combined cpDNA dataset of matK, rbcL, and trnL-F.

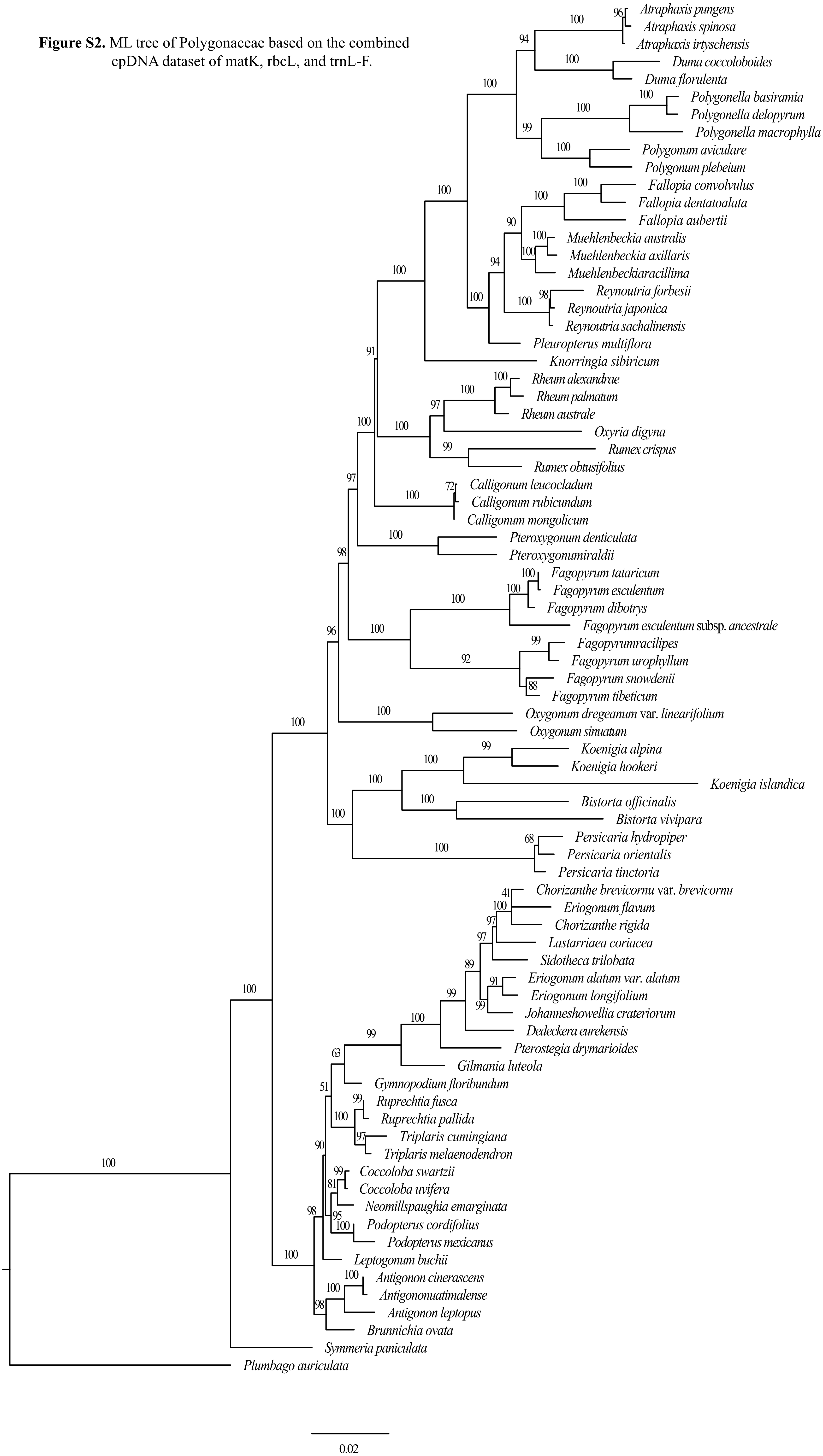

**Figure S3.** BI tree of *Fagopyrum* based on the combined cpDNA dataset of accD, matK, psbA-trnH, rbcL, and trnL-F.

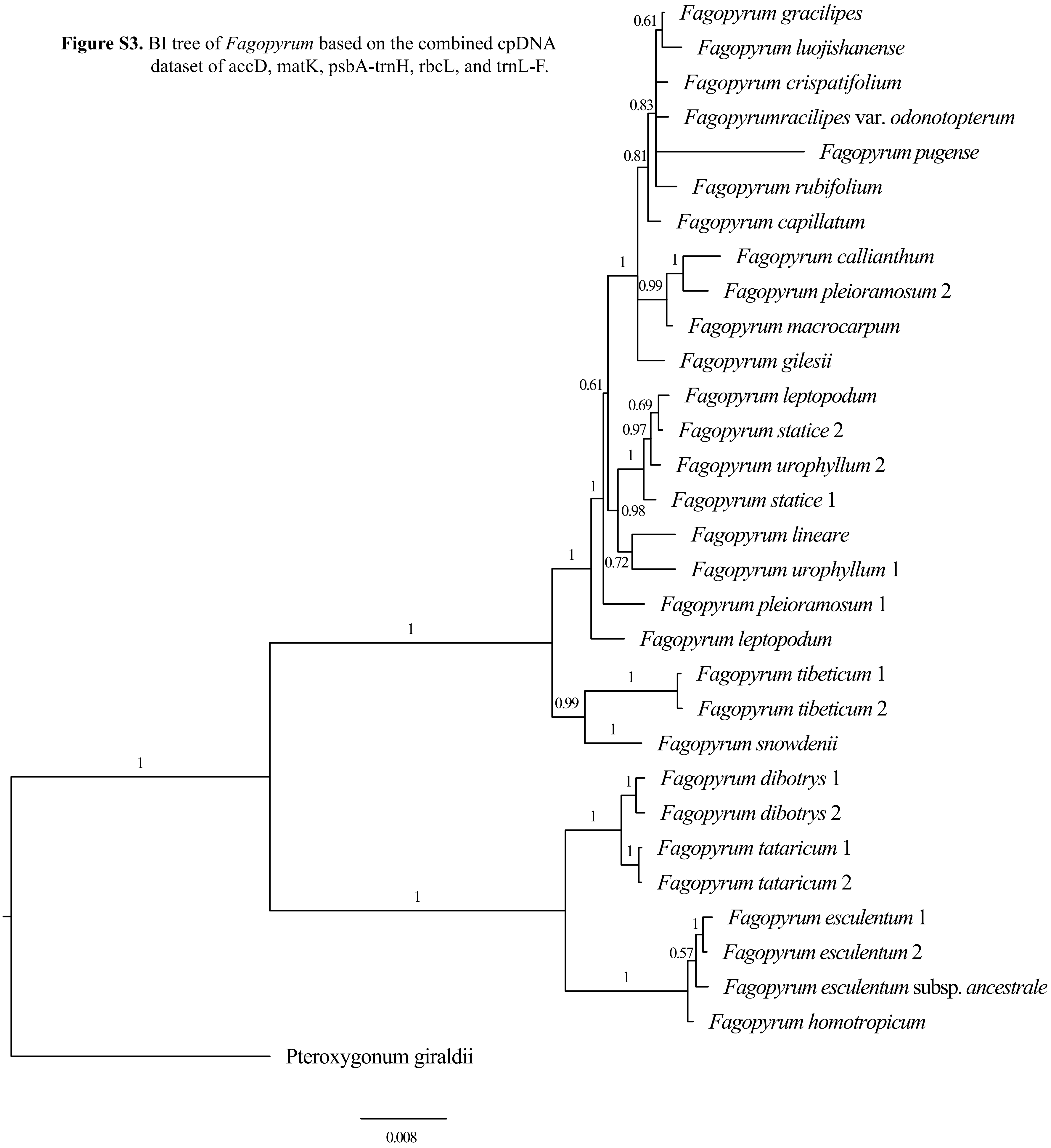

**Figure S4.** ML tree of *Fagopyrum* based on the combined cpDNA dataset of accD, matK, psbA-trnH, rbcL, and trnL-F.

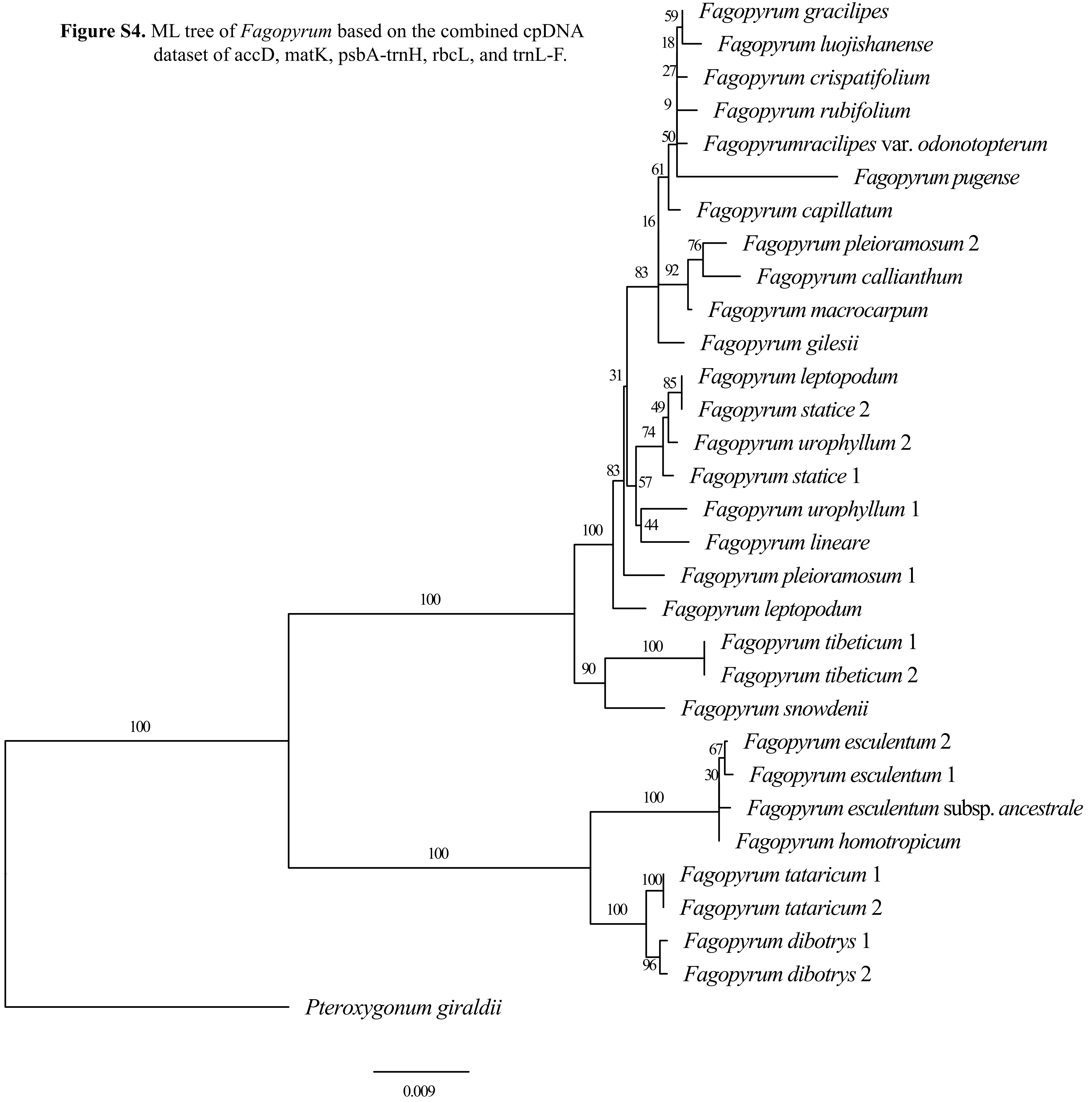

Supplement: Supplementary material 1 — Supplementary information [file phytokeys-220-109_article-97667__-s001.pdf]
